# Tubulointerstitial nephritis associated with primary biliary cirrhosis

**Published:** 2014-07-01

**Authors:** Mohammad-Hosein Rasolzadegan, Hanie Bakhshayesh, Nakisa Amid

**Affiliations:** ^1^Department of Nephrology, Yazd University of Medical Science, Yazd, Iran; ^2^Student Research Committee, Yazd Azad Islamic University of Medical Sciences, Yazd, Iran

**Keywords:** Tubulointerstitial nephritis, Anti-mitochondrial antibody, Primary biliary cirrhosis

**Implication for health policy/practice/research/medical education:**

Primary biliary cirrhosis (PBC) is an immune-mediated chronic cholestatic liver disease. In this paper, we report a patient with the rare association of tubulointerstitial nephritis and PBC.

Primary biliary cirrhosis (PBC) is an immune-mediated chronic cholestatic liver disease. The etiology is unknown, although it is presumed to be autoimmune in natural (1). PBC is most frequently a disease of women and occurs between the fourth and sixth decades of life. The symptoms may strongly affect patients’ quality of life and may induce incapacitation (2). The major pathology of this disease is a destruction of the small to median bile ducts, with leads to progressive cholestasis and often end-stage liver disease (3). Anti-mitochondrial antibody (AMA) can be found in 90–95% of patients with PBC, and they have a specificity of 98% for this disease. Biochemical pattern reveals cholestasis (elevation of alkaline phosphatase and γ-glutamyl-transferase; γ-GT), and the liver biopsy findings are compatible with bile duct injury, cholestasis and granulomas (4). Extra-hepatic associated disorders arise in 70% of patients with PBC (5). Tubulointerstitial nephritis is a primary injury to interstitial area and renal tubules, resulting in decreased renal function (6). The acute from is most often due to allergic drug reaction or to infection. The chronic from occurs with a diverse array of causes, including genetic or metabolic disorders, obstructive uropathy and chronic exposure to environmental toxins (7). Diagnosis is suggested by history and urinalysis and often confirmed by biopsy. In this paper, we report a patient with this rare association of tubulointerstitial nephritis (TIN) and PBC. Furthermore we evaluated clinicopathological features and outcomes in this patient.

A 28–year–old woman was admitted to our hospital with abdominal pain and jaundice. Pain has identified in right upper quadrant of abdomen and was progressive. Abdominal pain has continued since one year ago until now. There was excessive sweating, itching and anorexia. Patient had no drug history consumption, blood injection, using infected water or food in two years ago. On clinical examination blood pressure was normal. A one plus edema of the lower limbs was found and tenderness of right upper quadrant was detected. Cardiovascular examination and echocardiography were normal. Doppler sonography of the abdominal and lower limb vessels was normal, a non-obstructing renal stone in kidney was detected. Sonography showed upper normal limit of liver and some degree of liver coarseness. Gallbladder volume, echo and wall were normal and there was not any obstruction. Pancreas, spleen and biliary tract were normal. Kidneys showed mild decrease in size with increasing echotexture. Computed tomography of the abdomen showed length of right kidney and left was 9 cm and 8 cm respectively. Endoscopy of stomach was normal. All antigenic markers for malignancy were negative. C-reactive protein, rheumatic factor test, AMA and ANA was negative. Thyroid function tests were normal too. AMA-M₂ (IgM) and anti-CCP were 19.5 U/l and 35.3 U/l respectively. Proteinuria, glycosuria and hematuria were positive. Blood urea and creatinine levels were 73 mg/dl and 2 mg/dl respectively. Liver function tests were disturbed with aminotransferases at the upper normal range, AST; 185 U/l, ALT; 138 U/l, γ-GT; 698 U/l. Serology for hepatitis A, B and C were negative. Bilirubin level was normal. Intravenous cholangiography was negative. Β₂-macroglobulin was more than 4 mg/ml. In urine analysis, WBC; 8–10/hpf. ESR was 86 mm/1hr. Other biochemical tests were also within the normal limits. Liver biopsy showed inflammatory, well–formed granuloma surrounded by chronic inflammatory cellular infiltrate.

Kidney biopsy showed inflammatory cells mostly mononuclear type in interstitial area accompanied by tubular atrophy and interstitial fibrosis. Immunofluorescence study for IgA, IgG, IgM, C3, C1q and fibrin was not diagnostic.

The patient was discharged with the diagnosis of PBC with TIN. She was treated by ursodeoxycholic acid for liver disease and corticosteroid for deterioration of the renal function. Duration of two years follow up, general condition, serology of liver and renal function tests were improved and became stable up to report of the patient.

PBC is a chronic progressive cholestatic liver disease of unknown cause. Accumulated evidence suggests that cellular and humoral immunological abnormalities might cause the disease (8). An elevation of the ALT and AST may be identified in most patients with PBC, but significant elevations of ALP, γ-GT and immunoglobulin levels (mainly IgM) are usually the most prominent findings. The hallmark of this disease is the presence of AMA in the serum. Several extra-hepatic disorders closely associated with PBC are known, while renal involvement with TIN has rarely been described (9). PBC is rarely reported to be associated with tubulointerstitial nephritis, and rarely associated with other forms of renal disease too (10). Diagnosis is suggested by history and urinalysis and finally confirmed by renal biopsy. Treatment and prognosis vary by the etiology and potential reversibility of the disorder at the time of diagnosis. Differential diagnosis showed be made with other causes of cholestasis and especially with primary sclerosing cholangitis which is common in male and rarely associated with TIN (11,12). This case presented as cholestasis with unknown cause and liver function impairment that biochemical data suggested on associated renal disease. This form of presentation with PBC and TIN by a long period is also reported by others. In this patient, search for a causal relationship between TIN, PBC and other autoimmune disease (systemic lupus erythematosus, rheumatoid arthritis, grave’s disease and systemic vasculitis) or infections affecting the liver (hepatitis A, B or C), neoplasms and drug reactions was negative too. For the last two years, our patient maintained a stable renal and liver function profile. The disappearance of the urine abnormalities and the halting of the deterioration of the renal function, may be related to treatment by corticosteroid and an angiotensin-converting-enzyme inhibitor (ACE inhibitor). Renal and liver manifestations in this patient paralleled in severity of the disease, suggesting again a common probable autoimmune mechanism.

## Conclusion

PBC in patients with TIN is very rare and little is known about immunohistochemical characteristics of infiltrating cell in this setting.

## Authors’ contributions

MR and HB prepared the primary draft. NA prepared the final manuscript.

## Conflict of interests

The authors declared no competing interests.

## Ethical considerations

Ethical issues (including plagiarism, informed consent, misconduct, double publication and redundancy) have been completely observed by the authors.

## Funding/Support

None.
